# The effects of acute tryptophan depletion on instrumental reward learning in anorexia nervosa – an fMRI study

**DOI:** 10.1017/S0033291721005493

**Published:** 2023-06

**Authors:** Julius Steding, Franziska Ritschel, Ilka Boehm, Daniel Geisler, Joseph A. King, Veit Roessner, Michael N. Smolka, Florian Daniel Zepf, Stefan Ehrlich

**Affiliations:** 1 Translational Developmental Neuroscience Section, Division of Psychological and Social Medicine and Developmental Neurosciences, Faculty of Medicine, TU Dresden, Dresden, Germany; 2 Department of Child and Adolescent Psychiatry, Faculty of Medicine, University Hospital C. G. Carus, Technische Universität Dresden, Dresden, Germany; 3 Department of Psychiatry and Neuroimaging Center, Technische Universität Dresden, Dresden, Germany; 4 Department of Child and Adolescent Psychiatry, Psychosomatic Medicine and Psychotherapy, Jena University Hospital, Friedrich Schiller University, Jena, Germany; 5 Eating Disorder Treatment and Research Center, Department of Child and Adolescent Psychiatry, Faculty of Medicine, Technische Universität Dresden, Dresden, Germany

**Keywords:** Anorexia nervosa, serotonin, acute tryptophan depletion, reward, fMRI

## Abstract

**Background:**

The serotonin (5-HT) hypothesis of anorexia nervosa (AN) posits that individuals predisposed toward or recovered from AN (recAN) have a central nervous hyperserotonergic state and therefore restrict food intake as a means to reduce 5-HT availability (via diminished tryptophan-derived precursor supply) and alleviate associated negative mood states. Importantly, the 5-HT system has also been generally implicated in reward processing, which has also been shown to be altered in AN.

**Methods:**

In this double-blind crossover study, 22 individuals recAN and 25 healthy control participants (HC) underwent functional magnetic resonance imaging (fMRI) while performing an established instrumental reward learning paradigm during acute tryptophan depletion (ATD; a dietary intervention that lowers central nervous 5-HT availability) as well as a sham depletion.

**Results:**

On a behavioral level, the main effects of reward and ATD were evident, but no group differences were found. fMRI analyses revealed a group × ATD × reward level interaction in the ventral anterior insula during reward anticipation as well as in the medial orbitofrontal cortex during reward consumption.

**Discussion:**

The precise pattern of results is suggestive of a ‘normalization’ of reward-related neural responses during ATD in recAN compared to HC. Our results lend further evidence to the 5-HT hypothesis of AN. Decreasing central nervous 5-HT synthesis and availability during ATD and possibly also by dieting may be a means to normalize 5-HT availability and associated brain processes.

## Introduction

Anorexia nervosa (AN) is a psychiatric disorder characterized by a relentless pursuit of thinness and, despite being significantly underweight, an intense fear of gaining weight. Despite its high mortality rate (Erskine, Whiteford, & Pike, 2016) and significant global burden (i.e. in terms of disability-adjusted life years) the etiology of AN is still a subject of ongoing research (Zipfel, Giel, Bulik, Hay, & Schmidt, 2015).

One potentially promising neurobiological model of AN is focused on the neurotransmitter serotonin (5-HT). While diminished central nervous system availability of the essential amino acid tryptophan (TRP), the precursor for 5-HT synthesis, has been proposed as a risk factor for depressive symptoms (Cowen & Browning, 2015), several lines of research point to increased 5-HT availability as a risk factor for AN. To identify potential markers for AN risk, previous studies have investigated weight-recovered individuals with a history of AN (recAN; Frank, 2013). RecAN were shown to have increased cerebral spinal fluid (CSF) concentrations of the 5-HT metabolite 5-hydroxyindoleacetic acid (5-HIAA; Kaye, 1991; Kaye, Ebert, Raleigh, & Lake, 1984). Additional evidence comes from studies investigating monoamine oxidase activity (Ehrlich et al., 2008), platelet 5-HT content (Ehrlich et al., 2010) and cerebral 5-HT2 receptors (Frank et al., 2002) in recAN. Interestingly, an increased central nervous 5-HT synthesis rate seems to be also associated with anxiety symptoms, and dieting may be a way to reduce the intake of the TRP and subsequently lower (or potentially normalize) central 5-HT synthesis and 5-HT availability (Bailer & Kaye, 2010; Kaye et al., 2003; Kaye, Bailer, Frank, Wagner, & Henry, 2005a; Kaye et al., 2005b).

While, as described above, it has been hypothesized that premorbidly as well as after recovery from AN, (future/former) patients are in a hyperserotonergic state, i.e. they show an increased central nervous 5-HT availability, during the acute illness, the availability of TRP decreases due to restrictive eating. This may eventually ‘tip the scale’ toward a hyposerotonergic state, i.e. the central nervous 5-HT availability is decreased. Indeed, acutely underweight patients with AN have been shown to have reduced TRP plasma levels (Attia, Wolk, Cooper, Glasofer, & Walsh, 2005; Ehrlich et al., 2009; Gauthier et al., 2014) as well as reduced CSF concentrations of 5-HIAA (Demitrack, Heyes, Altemus, Pigott, & Gold, 1995; Kaye, Gwirtsman, George, Jimerson, & Ebert, 1988), a reduction in platelet imipramine (Weizman, Carmi, Tyano, Apter, & Rehavi, 1986) and paroxetine (Bruce, Steiger, Ng Ying Kin, & Israel, 2006; Ramacciotti, Coli, Paoli, Marazziti, & Dell'Osso, 2003) binding, a lower density of peripheral 5-HT2a receptors (Sigurdh, Allard, Spigset, & Hägglöf, 2013) and decreased central 5-HT2a receptor binding (Audenaert et al., 2003). Of note, dieting at the beginning of AN has been associated with (temporary) positive effects on mood and affect lability (Fitzsimmons-Craft et al., 2015; Selby et al., 2015). While a reduction of food intake leading to lower TRP availability and a subsequently reduced 5-HT synthesis in healthy controls (HCs) triggers hyperphagia (Burke & Heisler, 2015), patients with AN show continued low energy intake as well as low body weight.

A well-established experimental method to temporarily mimic changes of the central nervous 5-HT system is acute tryptophan depletion (ATD; Stewart et al., 2020). By ingesting mixtures of large neutral amino acids (LNAA) and high or low concentrations of TRP, the transport and availability of TRP and consequentially 5-HT synthesis in the brain can be modulated for several hours (Bell, Hood, & Nutt, 2005; Hood, Bell, & Nutt, 2005; Moja et al., 1988). ATD has been used to study the malleability of cognitive functions such as memory, attention and cognitive control as well as reward and emotional processing (Fusar-Poli et al., 2006; Kanen et al., 2021; Mendelsohn, Riedel, & Sambeth, 2009). It has also been employed to investigate 5-HT functioning in several psychiatric disorders, especially in mood and anxiety disorders (Bell, Abrams, & Nutt, 2001; Comai, Bertazzo, Brughera, & Crotti, 2020). In the only published ATD study with AN to date, recAN participants showed reduced anxiety levels (Kaye et al., 2003), supporting the hypothesis of a hyperserotonergic state as an antecedent of AN.

In addition to the aforementioned possible alterations in the central nervous 5-HT system, there is converging evidence for alterations in reward processing in AN (O'Hara, Campbell, & Schmidt, 2015). Most prominently, AN patients are able to suppress the drive to eat while being undernourished, but they also show anhedonic responses to other primary rewards such as sex and pleasant touch (Bischoff-Grethe et al., 2018; Crucianelli et al., 2020; Raboch & Faltus, 1991) and in general a reduced sensitivity to reward in self-report studies (Harrison, O'Brien, Lopez, & Treasure, 2010). With respect to neural correlates, study results have shown altered neural responses to disorder-related (e.g. food) as well as disorder-unrelated (e.g. money) stimuli in typical regions of the brain reward system such as the ventral striatum (VS; Cowdrey, Park, Harmer, & McCabe, 2011; Decker, Figner, & Steinglass, 2015; Fladung *et al*. 2010; Frank *et al*. 2012; Wagner *et al*. 2008; Wierenga *et al*. 2015) and the medial orbitofrontal cortex (mOFC; Frank et al., 2012; Uher et al., 2003).

While dopamine (DA) has long been regarded as the main neurotransmitter system involved in reward processing, a growing number of studies using experimental manipulations of 5-HT synthesis in humans have implicated the 5-HT system to have effects on reward (and punishment) processing (Cools, Roberts, & Robbins, 2008; Crockett, Clark, & Robbins, 2009; Evers et al., 2005; Schweighofer et al., 2008; Seymour, Daw, Roiser, Dayan, & Dolan, 2012). Depending on the 5-HT receptor subtype, 5-HT can enhance or inhibit the dopaminergic effects in the brain reward system (Cools, Nakamura, & Daw, 2011; Daw, Kakade, & Dayan, 2002; Di Matteo, Di Giovanni, Pierucci, & Esposito, 2008). In animal studies, there is evidence that the additive effect of DA and 5-HT results in a strongly rewarding sensation, whereas each of the neurotransmitters alone does not yield the same effects (Fischer & Ullsperger, 2017). Taken together, these results dovetail with a prominent theory that posits that the reward system is also modulated by the 5-HT system (Cools et al., 2011). According to this view, a possible underlying computational mechanism may include that decreasing 5-HT availability increases the expected (average) outcome and thereby modulate computational parameters of reward processing and reward-based decision-making. Although the literature on the neural correlates of reward processing during 5-HT manipulations in humans is overall relatively sparse, several pharmacological fMRI studies using ATD and selective serotonin reuptake inhibitors (SSRI) have shown blood oxygen level-dependent (BOLD) signal changes in regions typically involved in reward processing such as the VS, the OFC as well as the insula (for a review, see Macoveanu, 2014).

In order to probe serotonergic modulation of reward-related neural responses in the context of AN, the current double-blind crossover study used ATD in combination with an established instrumental motivation task (Bühler et al., 2010; Ehrlich et al., 2015) using monetary rewards during functional magnetic resonance imaging (fMRI) in a sample of recAN and carefully age-matched HC. We hypothesized that a hyperserotonergic state is an underlying risk factor in the pathogenesis of AN, which is (temporarily) lowered by food restriction at the onset of the disorder but, due to severe emaciation, deteriorates into a hyposerotonergic state in the acutely underweight state of the disorder. Therefore, an experimental reduction of 5-HT via ATD should ‘normalize’ altered brain responses to reward in recAN individuals, for which purpose we focused on three-way interactions of ATD condition, group and reward in the BOLD signal.

## Methods

### Sample description

For this study, we recruited a total of 49 female participants. The final sample consisted of 47 individuals (*n* = 22 recAN and *n* = 25 HC) who completed both study sessions (i.e. ATD and sham depletion). Two HC dropped out between the two sessions (*n* = 1 due to subjective intolerance of the ATD procedure and *n* = 1 due to scheduling difficulties). To be considered ‘recovered’, participants had to (1) maintain a body mass index (BMI) of >18.5 (age 19 to 29) or be above the 10^th^ BMI percentile with respect to their age (age 12 to 18; Kromeyer-Hauschild et al., 2001) for at least 12 months, (2) menstruate, and (3) refrain from binging, purging or other substantially restrictive eating patterns. The control group consisted of normal-weight, eumenorrhoeic women with no history of psychiatric illness. We generally excluded participants if they had a lifetime history of any of the following clinical diagnoses: bulimia nervosa or binge eating disorder, schizophrenia, substance dependence, bipolar disorder and suicidality. Further exclusion criteria for all participants were incomplete ATD drink intake, nausea or other related intolerance of the ATD mixture, IQ lower than 85, organic brain syndrome, chronic medical or neurological illnesses that could affect appetite, eating behavior or body weight (e.g. diabetes), pregnancy, breast feeding, use of psychotropic medications or substances within the 4 weeks preceding the study. See Supplemental Material 1 (SM 1) for a full list of exclusion criteria.

This study was approved by the ethics committee of the Technische Universität Dresden and carried out in accordance with the latest version of the Declaration of Helsinki, and all participants (and their guardians if underage) gave written informed consent.

### Clinical measures

For all participants, the presence or absence of current diagnoses of eating disorders was ascertained by evaluation of the expert form of the Structured interview of AN and bulimia nervosa (SIAB-EX; Fichter & Quadflieg, 1999). Additionally, a structured clinical interview (Mini-DIPS; Margraf, 2013) was used to rule out other active psychiatric disorders. Interviews were conducted by clinically experienced and trained research assistants under the supervision of the attending child and adolescent psychiatrist.

In addition to the clinical interviews, eating disorder-specific psychopathology was assessed with the German version of the Eating Disorders Inventory (EDI-2; Paul & Thiel, 2005). Furthermore, depressive symptoms were examined using the Beck Depression Inventory II (BDI-II; Hautzinger, Kühner, & Keller, 2009). IQ was assessed with a short version of the German adaption of the Wechsler Adult Intelligence Scale (von Aster, Neubauer, & Horn, 2006). BMI standard deviation scores (BMI-SDS) were calculated for each participant, which is controlled for both age and sex (Kromeyer-Hauschild et al., 2001).

### Procedure

The ATD procedure entailed a double-blind, randomized crossover design with each participant undergoing both the depletion and the sham depletion within a period of 7–14 days to avoid potential carry-over effects (*M* = 9.64, s.d. = 3.21). On the day prior to the study appointments, participants were asked to restrict meals to low protein food and given a list with meal suggestions. Immediately before the ATD and sham depletion intake at 8.30 a.m., participants were weighed. The ATD and sham depletion drinks were then prepared using a weight-adapted dosing regimen (Moja et al., 1988; Zepf et al., 2014; for a more detailed description of the ATD mixture, see the Supplements 2). After the ATD/sham depletion intake, participants were given a TRP free breakfast. 4 h after the ATD/sham depletion intake, the MRI session was performed. Participants were continuously supervised by trained staff.

### Biochemical measures

Blood samples were taken before the ATD/sham depletion intake as well as approximately 5 h later immediately after the MRI session using a venous catheter (Braunüle®) and ethylenediaminetetraacetic (EDTA) tubes (Sarstedt, Germany). For more details on the biochemical measures and analyses, see SM 3.

To quantify the depletion effect, the ratio of plasma TRP to LNAAs (namely valine, methionine, isoleucine, leucine, phenylalanine, thyrosine) was calculated for each participant using the values of the blood samples immediately after the MRI session (5 h after ATD or sham depletion intake).

### Instrumental motivation task

**Fig. 1.**
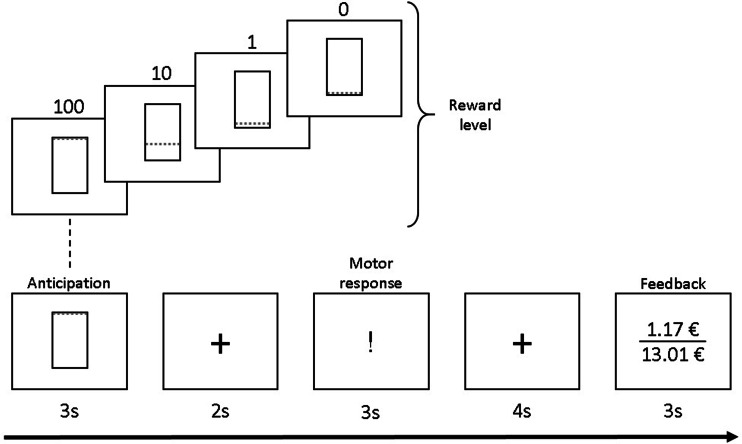
Instrumental motivation task. Instrumental motivation task during event-related functional MRI (fMRI). During the anticipation phase a visual cue was presented for 3 s to inform the participant about the reward level of this trial [reward levels: 0 (no reward), 1, 10, 100]. The motor (or instrumental) response phase started after a 2 s fixation period. Monetary reward per trial increased with reward level and higher effort and was determined by multiplying number of button presses × reward level × an individual adjustment factor (calculated based on the individual maximum #bp in the test run; for details see Bühler et al., 2010). Acoustic feedback for button presses was provided through headphones. After another fixation period of 4 s, feedback was provided for 3 s by displaying the amount of money gained in this trial and the cumulative amount. Between trials, participants fixated on crosshairs for 3 s (75%) or 7.44 s in 25% of all trials, which improves design efficiency by jittering. The fMRI main run had a total duration of 15.5 min and comprised 48 trials in total (4 reward levels × 12 pseudorandomized repetitions).

During the fMRI session, participants performed the same instrumental motivation task (Bühler et al., 2010; Kroemer et al., 2014) that we have used in previous studies in both acute (Steding et al., 2019) and weight-recovered (Ehrlich et al., 2015) AN samples. In addition to allowing for measurement of event-related brain activity in response to stimuli predicting monetary reward (reward anticipation) and feedback about the magnitude of the reward received, this particular task variant has the advantage of providing behavioral assessment of motivation operationalized as instrumental responding (number of button presses, #bp) to maximize reward. Each trial included an anticipation phase, a motor response phase, and a feedback (receipt) phase (Fig. 1 for details). The scanning session started with an eight-trial test run to determine each individual's maximum #bp. This information was used to standardize the cumulative monetary gain to ≈ €10 in the subsequent main run, irrespective of inter-individual performance differences in motor speed (for more information, see SM 4).

### Instrumental response data analysis

We compared average #bp and reaction times (RT) of initial responses at each reward level between recAN and HC participants using linear mixed-effects models for the analysis of repeated measurement treating participants as random effects. We assumed a compound symmetry covariance structure for changes of instrumental response by reward level (0, 1, 10, 100) and included group (recAN, HC) and individual TRP/LNAA ratios as indicators of the ATD condition as factors as well as interaction effects (slope) between all variables. Since the variable indicating reward level was centered (and HC were used as a reference for the factor group) the intercept in HC (and intercept + main effect of group in recAN) represents not instrumental responses at reward level 0 but the ‘typical’ response, i.e. it correlates highly with the response rate at an average reward level.

### Structural and functional image acquisition, data processing and analysis

Images were acquired using standard sequences with a 3 T whole-body MRI scanner (TRIO; Siemens, Erlangen, Germany) equipped with a standard head coil (SM 5). Functional and structural images were processed with SPM8 (http://www.fil.ion.ucl.ac.uk/spm) within the Nipype framework (http://nipy.sourceforge.net/nipype/; Gorgolewski, Storkey, Bastin, & Pernet, 2012) following standard procedures (SM 6). We evaluated fMRI data quality by manual inspection and using the artifact detection tool (ART; Whitfield-Gabrieli et al., 2009). Volumes that exceed an intensity threshold of three standard deviations or a threshold of 2 mm normalized movement in any direction were classified as outliers and excluded from statistical models (motion-outlier: recAN individuals, day 1: 2.47 ± 10.31, day 2: 0.35 ± 0.87; HC, day 1: 0.05 ± 2.13, day 2: 0.18 ± 0.66; intensity-outlier: recAN individuals, day 1: 6.84 ± 6.87, day 2: 7.75 ± 6.23; HC, day 1: 5.27 ± 4.47, day 2: 8.14 ± 6.66).

On the single-subject level, a general linear model (GLM) was fit to model the hemodynamic response to increasing reward levels. We modeled all four reward levels of the anticipation, motor response and feedback phase as single events (12 regressors). Additional regressors included six motion parameters as well as one regressor for each motion or intensity outlier volume (see SM 6).

To test our hypothesis regarding the effect of ATD on reward processing in AN in comparison to HC with second-level whole-brain analyses, we used neuropointillist (http://ibic.github.io/neuropointillist/; Madhyastha et al., 2018) to fit linear mixed models in R Studio (nlme package) to each voxel for each participant separately for the anticipation and feedback phase during both time points. The mixed model to investigate the three-way interaction of ATD, group and reward comprised the four centered reward levels, group as well as individual TRP/LNAA ratios as indicators of the ATD condition. This procedure then yields statistical parameter maps for each main and interaction effect. To control for false-positives, family-wise error (FWE) correction was performed using 3DClustSim (http://afni.nimh.nih.gov/afni; ‘fixed’ version compiled June 2017). Afterward, to aid interpretation of significant whole-brain results and since neuropointillist only yields statistical parameter maps, we obtained extracted beta values averaged from significant clusters of three-way interaction effects using the MarsBaR toolbox for SPM (Brett, Anton, Valabregue, & Poline, 2002) and ran the same linear mixed models (in a post-hoc approach) with the extracted indices using R Studio (nlme package).

## Results

### Sample characteristics

There were no significant differences between the groups regarding age, IQ, BMI, BMI-SDS as well as BDI-II. However, AN symptoms as measured by EDI-2 were higher in the recAN group compared to the HC (Table 1).

**Table 1.** Demographic and clinical characteristics of the sample

	recAN (*n* = 22)		HC (*n* = 25)		*t*	*p*
	*M*	s.d.	*M*	s.d.		
Age	23.03	2.83	22.96	2.48	0.08	0.94
Recovery duration (months)	55.73	34.42	–	–	–	–
Duration of illness (years)	3.43	2.42	–	–	–	–
IQ	111.23	8.41	111.64	7.93	0.17	0.86
BMI	21.03	1.48	21.06	2.02	0.52	0.96
BMI-SDS	−0.43	0.52	−0.46	0.63	0.18	0.86
EDI-2 total	161.5	46.64	123.4	17.59	3.61	0.001
BDI-II	6.18	8.19	2.44	3.27	2.01	0.055

*Notes.* All data of this table refers to day 1 of the study. recAN = Individuals recovered from anorexia nervosa, HC = healthy control participants. Group differences were tested using Student's *t* tests. IQ = intelligence quotient, BMI = body mass index, BMI-SDS = body mass index standard deviation score, EDI-2 = Eating Disorder Inventory 2, BDI-II = Beck Depression Inventory-II. For time since recovery, values ranged from 12 to 140 months. Eighteen recAN individuals were of the restrictive subtype and four of the binge/purge subtype. For duration of illness, the values for *n* = 4 recAN participants were not obtainable; values ranged from 0.5 to 9.8 years.

### Plasma tryptophan levels

Demonstrating a successful tryptophan manipulation in both groups, tryptophan levels were significantly lower during the ATD condition than during the sham depletion (*F*_(1, 44)_ = 334.4, *p* < 0.0001; Figure S.1 in SM 7).

### Instrumental response data

**Fig. 2.**
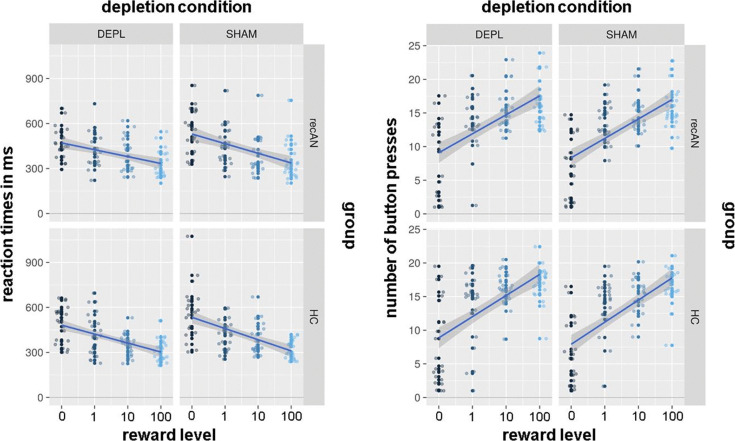
Behavioral data of both groups during both conditions. Each dot represents the mean value of one participant for each reward level (0, 1, 10, 100). The left panel shows the distribution of reaction times and the right one the number of button presses (plus smooth regression lines). DEPL = acute tryptophan depletion; SHAM = sham depletion; recAN = individuals with a history of AN; HC = healthy control participants.

As in previous studies with the employed paradigm (Bühler et al., 2010; Ehrlich et al., 2015; Steding et al., 2019), RTs decreased (*F*_(1, 312)_ = 70.02, *p* < 0.0001) and #bp increased (*F*_(1, 312)_ = 104.48, *p* < 0.0001) with ascending reward level as expected, but no significant group differences were evident (for both *F*_(1, 44)_<0.7, n.s.). For #bp, there was a significant main effect for the ATD treatment (*F*_(1, 312)_ = 13.47, *p* < 0.001) with higher #bp values during the depletion condition (*M* = 13.4, s.d. = 2.88; sham depletion: *M* = 12.75, s.d. = 2.3). For both behavioral measures, none of the interactions were significant. For more details, see Fig. 2 as well as Table S.1 in SM 8.

### Neuroimaging data

#### Anticipation phase

**Fig. 3.**
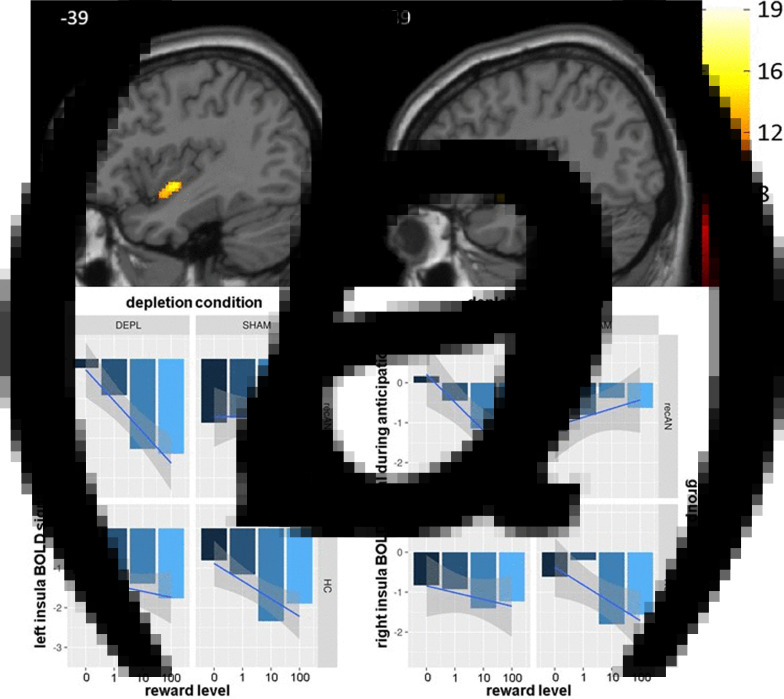
(*a*) Results of the whole-brain voxelwise mixed model of the reward anticipation phase. Left: significant three-way interaction of group × depletion condition × reward level within the left ventral anterior insula (*x* = −39). Right: significant three-way interaction of intervention × group × reward level within the right ventral anterior insula (*x* = 39). FWE corrected with *p* < 0.05. (*b*) Mean activation in each group in each reward level (*β* estimates plus a smooth regression line) in the left and right ventral anterior insula during the reward anticipation phase at both time points (ATD and sham depletion). DEPL = acute tryptophan depletion; SHAM = sham depletion; recAN = individuals with a history of AN; HC = healthy control participants; BOLD = blood oxygen level dependent.

As a proof of concept, we first investigated the main effect of reward level during reward anticipation and found a parametric linear increase in both the left and the right *v.* (see Fig. 3) as in our previous studies (Ehrlich et al., 2015; Steding et al., 2019)**.** Regarding our motivating hypothesis of a group difference in the effect of ATD on reward processing, we uncovered significant results in our mixed models in both the left (peak voxel at *x* = −38, *y* = −4, *z* = −8; *F* = 15.41) and the right (*x* = 38, *y* = 4, *z* = −14; *F* = 16.02) ventral anterior insula during reward anticipation (three-way interaction of group × depletion condition × reward level; Figure 3). BOLD parameter estimates extracted from the identified bilateral clusters revealed a significant main effect of reward level (left: *F*_(1, 319)_ = 27.94, *p* < 0.0001; right: *F*_(1, 323)_ = 10.75, *p* = 0.001) in addition to confirming the aforementioned three-way interaction (left: *F*_(1, 319)_ = 22.80, *p* < 0.001; right: *F*_(1, 323)_ = 15.24, *p* < 0.001; Figure 3) as expected. Post-hoc *t* tests of the reward-related BOLD signal slopes suggested that the recAN group during the sham depletion is the main factor contributing to the three-way interaction (Table S.2 in SM 1). More specifically, the BOLD signal slopes of the recAN group during the sham depletion condition were significantly flatter (i.e. less negative) compared to the slopes of the HC group in the same condition as well as compared to the same recAN group during the ATD condition. Additionally, recAN during ATD showed no significant difference compared to HC during the sham condition. Taken together, this could be an indication of an ATD-dependent normalization of BOLD responses in former AN patients.

#### Feedback phase

**Fig. 4.**
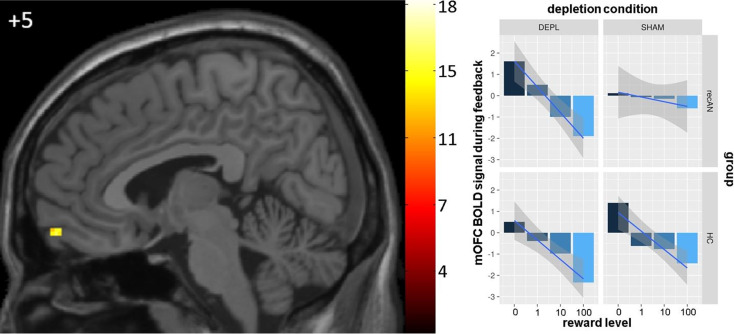
Results of the whole-brain voxelwise mixed model of the reward feedback phase. Left: Significant three-way interaction of group × depletion condition × reward level within the mOFC (*x* = 5). FWE corrected with *p* < 0.05. Right: Mean activation in each group for each reward level (*β* estimates plus a smooth regression line) in the mOFC during the reward feedback phase during both time points (ATD and sham depletion). mOFC = medial orbitofrontal cortex; DEPL = acute tryptophan depletion; SHAM = sham depletion; recAN = individuals with a history of AN; HC = healthy control participants; BOLD = blood oxygen level dependent.

We also found a group difference in the effect of ATD on reward processing during feedback (three-way interaction) in our mixed model analyses in the mOFC (peak voxel at *x* = 4, *y* = 60, *z* = −16; *F* = 15.16; Fig. 4). BOLD parameter estimates extracted from the identified cluster revealed a main effect of reward level (*F*_(1, 323)_ = 82.23, *p* < 0.0001) and a depletion condition × reward level interaction (*F*_(1, 323)_ = 7.74, *p* = 0.006) in addition to confirming, as expected, the aforementioned three-way interaction (*F*_(1, 323)_ = 16.06, *p* = 0.0001; Fig. 4). Similar to the anticipation phase, post-hoc *t* tests of the reward-related BOLD signal slopes suggested that the recAN group during the sham depletion is the main factor contributing to the three-way interaction, thus indicating that under ATD, reward-related BOLD responses seem to normalize in former AN patients (Table S.3 in SM 11).

#### Exploratory brain behavior correlations

Given that the observed group differences during reward anticipation and consumption are seemingly attributable to the abnormal activation in recAN during sham depletion, we explored potential brain-behavior relationships between recAN group activation under this condition (slope of each participant) and age, BMI-SDS, EDI-2 total score as well as BDI-II. However, there were no significant correlations (for more detailed information, see Table S.4 in SM 12).

## Discussion

In this fMRI study, we investigated the influence of an experimental modulation of central nervous 5-HT availability (ATD) on reward processing in weight-recovered women with a history of AN. While there were only group-independent effects of ATD on behavioral responses, we found a three-way interaction of the group, ATD condition and reward level in both the bilateral ventral anterior insula during the reward anticipation phase as well as in the mOFC during the feedback phase. This pattern of results was mainly attributable to BOLD responses in recAN participants during sham depletion. In the depletion condition, however, recAN individuals showed BOLD signal slopes similar to those of HC during depletion and the sham depletion. Based on the 5-HT hypothesis of AN that AN-related food restriction mitigates a hyperserotonergic state (Kaye et al., 2003, 2005b), one possible interpretation of these findings is that ATD reduces (potentially ‘normalizes’) hyperserotonergic functioning in recAN.

On the behavioral level, we found the main effect of ATD treatment on instrumental responses indicating higher #bp in the depletion relative to sham condition. This is in line with previous studies reporting generally faster response speed and relative disinhibition after ATD (Cools et al., 2011; Crockett et al., 2009). Although we did not find a faster average RT of the initial response, potentially due to floor effects, the general increase in average #bp is indicative of faster responding for the following button presses. This supports the notion of a behavioral disinhibition effect of tryptophan depletion (Walderhaug et al., 2002), even though it has been proposed to be more prominent in response to aversive stimuli and punishment (Faulkner & Deakin, 2014).

Our neuroimaging findings in the insula and mOFC fit well with several previous studies using manipulations of 5-HT availability which have reported alterations in both of these regions (for a review, see Macoveanu, 2014). For example, Seymour and colleagues (Seymour et al., 2012) found an interaction of ATD and reward indicating a dependence of the reward outcome value on 5-HT signaling in both the mOFC as well as the anterior insula in healthy participants performing a probabilistic instrumental learning task. The (anterior) insula has long been overlooked in the reward literature despite being connected to limbic regions such as the mOFC, *v.* and the amygdala (Sescousse, Caldú, Segura, & Dreher, 2013). It is this interconnectedness that led to the hypothesis of the insula being one of the most relevant brain regions with respect to the psychopathology as well as the neuropsychological and neural alterations regularly observed in AN patients (Nunn, Frampton, Gordon, & Lask, 2008). For example, studies investigating sensory-interoceptive reward signals in individuals recovered from AN (recAN) found altered anterior insula activation in response to sweet taste (Oberndorfer et al., 2013a; Wagner et al., 2008) and food images (Oberndorfer et al., 2013b).

In line with the hypothesis that vulnerability toward AN may be associated with a hyperserotonergic state (Bailer & Kaye, 2010; Kaye et al., 2003, 2005a, 2005b), we found altered BOLD signal patterns during sham depletion but an apparent relative normalization of neural activity in the ventral anterior insula as well as the mOFC in recAN following ATD. In accord with this account, pharmacological studies using SSRIs to treat AN failed to show significant clinical effects on both body weight and mood (for a review, see Frank, 2020); potentially because pharmacologically increasing 5-HT availability in the brain may worsen a pre-existing hyperserotonergic state. This is consistent with the finding by Kaye et al. (2003) of an anxiolytic effect in recAN compared to HC following an ATD intervention which laid the foundation of the 5-HT hypothesis in AN. This may explain why AN patients report dysphoric mood with increasing food intake during and after treatment (Frank & Kaye, 2012) as well as positive effects on mood (Fitzsimmons-Craft et al., 2015) and positive affect lability (Selby et al., 2015) in association with restrictive eating. Interestingly, the absence of significant correlations in our exploratory analyses of brain-behavior relationships of the clinical markers in recANs during sham depletion hints toward the presence of a trait marker in recAN since the neural responses were not related to residual symptoms. Taken together, the 5-HT hypothesis could be of interest with respect to clinical implications. For example, pharmacological agents that lower or modulate 5-HT such as 5-HT antagonists could prove to be useful in treating AN. However, more research is needed since several of already existing drugs are not only antagonists to 5-HT, but also to other neurotransmitters such as dopamine and histamine which makes causal conclusions difficult (Frank, 2020). Additionally, this research must be extended to other eating disorders in which the central 5-HT system has been implicated as well such as bulimia nervosa (Kaye et al., 2000).

The interpretation of the findings of this study should be seen in the context of several limitations. Although a within-subject design has greater statistical power than between-subject designs and may thus detect effects with smaller samples, future studies with larger samples are needed to replicate and confirm our results. It should also be noted that although monetary rewards enable generalization with respect to reward processing, they tend to show mixed results in AN samples (Haynos, Lavender, Nelson, Crow, & Peterson, 2020) and additional studies using other rewards (e.g. positive social feedback) might be particularly informative to the 5-HT hypothesis. Furthermore, while being widely used and validated for both adults and adolescents (for a review, see Stewart et al., 2020), the ATD intervention may have certain weaknesses. The success and the impact of ATD on brain 5-HT availability is judged by plasma TRP/LNAA ratios, which is only an indirect measure. Accordingly, conclusions about selective serotonergic effects should be made with caution (van Donkelaar et al., 2011; but see also the response by Crockett et al., 2012; as well as this review Young, 2013). However, Nishizawa and colleagues have measured the actual influx of TRP into the brain after ATD using PET and confirmed its effectiveness (Nishizawa et al., 1997) and Ardis et al. (2009) reported evidence for a relatively selective serotonergic effect of ATD since it did not alter concentrations of other monoaminergic neurotransmitters. Regarding the definition of AN recovery, the lack of a broad consensus has been criticized. In our sample, one of the inclusion criteria was the resumption of mensis and the duration of recovery exceeded the recommended time period of 12 months (mean recovery time of 55.73 months; Wade & Lock, 2020). Lastly, with respect to the notion that a hyperserotonergic state might be relevant in the pathogenesis of AN, it should be noted that with the design of this study, it can not be concluded whether this is a trait or a scar effect (Frank, 2013; Seidel et al., 2020).

To conclude, this study adds further evidence supporting the 5-HT hypothesis of AN (Bailer & Kaye, 2010; Kaye et al., 2003, 2005b) by showing an apparent ‘normalization’ of neural patterns (BOLD signal) in the ventral anterior insula and the mOFC during reward processing in individuals recAN during ATD. Since this is the first study that investigated reward processing in AN in combination with ATD, further studies are needed to confirm our findings (Horster et al., 2020). If confirmed, such a mechanism might help to explain why dieting is often reported as increasing the subjective well-being (at least initially) of individuals with or at risk for AN (Miyasaka et al., 2003). Furthermore, these findings also have clinical implications. For example, treatment approaches using pharmacological agents such as (partial) serotonin antagonists may be an interesting avenue for future research with the aim to balance out potentially increasing 5-HT availability during refeeding (Frank & Shott, 2016).

## Supplementary material

For supplementary material accompanying this paper visit https://doi.org/10.1017/S0033291721005493. click here to view supplementary material
