# Supplementary material for: The effects of acute tryptophan depletion on instrumental reward learning in anorexia nervosa – an fMRI study
Source: Psychol Med. 2022 Mar 28;53(8):3426–36. doi: 10.1017/S0033291721005493 (PMC10277771; doi:10.1017/S0033291721005493)
Supplement: Supplementary file 1 [file S0033291721005493sup.zip › S0033291721005493sup002.docx]

**The effects of acute tryptophan depletion on instrumental reward learning in anorexia nervosa - an fMRI study**

***Supplementary Material***

Julius Steding, Franziska Ritschel, Ilka Boehm, Daniel Geisler, Joseph A. King, Veit Roessner, Michael N. Smolka, Florian Daniel Zepf, Stefan Ehrlich

## 1. Exclusion criteria

As in our previous studies that focused on weight-recovered individuals with a history of anorexia nervosa (recAN; Ehrlich et al., 2015; King et al., 2019), participants of both groups were excluded if they reported current selective serotonin reuptake inhibitors (SSRI) intake, psychotropic drug intake within the last 4 weeks, being pregnant or breast feeding, anemia, being younger than 12 or 30 and older, having an IQ below 85, suffering from organic brain syndrome, dementia, schizophrenia, bipolar disorder, drug abuse, obesity (BMI >97th percentile for age <18, BMI >30 for age 18 and older) as well as chronic medical or neurological illnesses that could affect appetite, eating behavior or body weight (e.g. diabetes).

As for the healthy control participants (HC), they were excluded if they had a lifetime diagnosis of any psychiatric disorder, the lowest lifetime BMI below the 10th percentile (below age 18) or <17.5 (for age 18 and older) or if they were currently underweight, showed abnormal eating behavior (diet, binge eating) or a binge eating disorder.

Additionally, recAN were excluded if they lifetime diagnosis of atypical AN, bulimia nervosa or binge eating disorder.

## 2. ATD mixture

The dose of amino acids in the experimental mixtures were adjusted to the weight of each participant (Dingerkus et al., 2012; Moja et al., 1988; Zepf et al., 2014). Both mixtures contained the same amount of large neutral amino acis (LNAA), but differed in their tryptophan levels. Accordingly, tryptophan was completely absent in the mixture of the ATD condition, while the mixture of the sham depletion contained 7mg/kg body weight (recommended daily dose for adults; Richard et al., 2009). The dose of large neutral amino acids (LNAA) was constant for every participant across sessions: L-phenylalanine (132 mg/kg), L-leucine (132 mg/kg), L-isoleucine (84 mg/kg), L-methionine (50 mg/kg), L-valine (96 mg/kg), L-threonine (60 mg/kg), and L-lysine (96 mg/kg).

Participants were informed about the bad taste of the mixtures and instructed to best drink it all at once. If wished for, they could drink any other tryptophan free drink afterwards.

## 3. Biochemical measures

Immediately after taking the blood samples, they were centrifuged at 4,000g and 4°C for 10 minutes. Afterwards, the plasma was stored in a -81°C fridge. Finally, blood analyses were conducted at the Institute for Clinical Chemistry and Laboratory Medicine of the Technische Universität Dresden, Medizinische Fakultät. The analyses of physiological amino acids were performed by cation exchange chromatography with post-column derivatization, using the Biochrome amino acid analyzer B30 (http://www.biochrom.co.uk). To avoid influences of calibration, all samples from each patient were run within one and the same series, using additional internal standard in each sample.

Missing values (n=3 during the first session, n=5 during the second) due to complications during blood sampling were imputed by their means per group and ATD condition.

## 4. Instrumental motivation task

This task variant also provides behavioral assessment of motivation operationalized as instrumental responding to maximize reward (Bühler et al., 2010; Kroemer et al., 2014). Instrumental responding is measured via the number of button presses and reaction times (RT) in the motor (or instrumental) response phase of the task. RT was defined as the time elapsing between the beginning of the appearance of the visual stimulus (exclamation mark) and the beginning of a participant’s reaction to it (first button press) in milliseconds.

Before the scanning session, participants completed a practice version of the task (12 trials) to learn how to perform it.

The raw behavioral data can be found in the supplementary table “behavioral_data.xlsx”.

## 5. Structural and functional image acquisition

Structural and functional MRI data were acquired with a 3T scanner (Magnetom Trio, Siemens). The T1-weighted structural brain scans were acquired with rapid acquisition gradient echo (MP-RAGE) sequence with the following parameters: number of slices=176; repetition time (TR) 1900 ms; echo time (TE) 2.26 ms; flip angle 9°; slice thickness 1 mm; voxel size 1 × 1 × 1 mm³; field of view (FOV) 256 × 224 mm²; bandwidth 200 Hz/pixel. The functional images were acquired using gradient-echo T2*-weighted echo planar imaging (EPI) with the following parameters: tilted 17° from anterior–posterior commissure line toward coronal (to reduce signal dropout in orbitofrontal regions); number of volumes=396; number of slices=42; TR 2410 ms; TE 25 ms; flip angle 80°; 3 mm inplane resolution; slice thickness 2 mm (1 mm gap resulting in a voxel size of 3 × 3 × 2 mm³); FOV 192 × 192 mm²; bandwidth 2112 Hz/pixel.

## 6. Functional image data processing and analysis

The slice time corrected functional EPI data were 4D-realigned and registered to their mean. The 6 realignment parameters, characterizing the rigid-body movement (x,y,z, pitch, roll, yaw), were saved and later used as nuisance covariates to account for the variance due to motion. Subsequently these images were coregistered to the participant’s structural brain image. A DARTEL template was created using structural images from all participants (Ashburner, 2007). The EPI volumes were then normalized to Montreal Neurological Institute space using the DARTEL template and corresponding flow field. The resulting data were smoothed with an isotropic 8 mm full-width at half-maximum Gaussian kernel. During the image data processing at the single participant-level using a general linear model (GLM), all regressors were convolved with a synthetic hemodynamic response function as implemented in SPM8. Cluster thresholds for the regions identified in our neuropointillist analyses as calculated using 3dClustSim were 16 mm³ for both the left and the right anterior ventral insula and 46 mm³ for the mOFC.

## 7. Plasma tryptophan availability


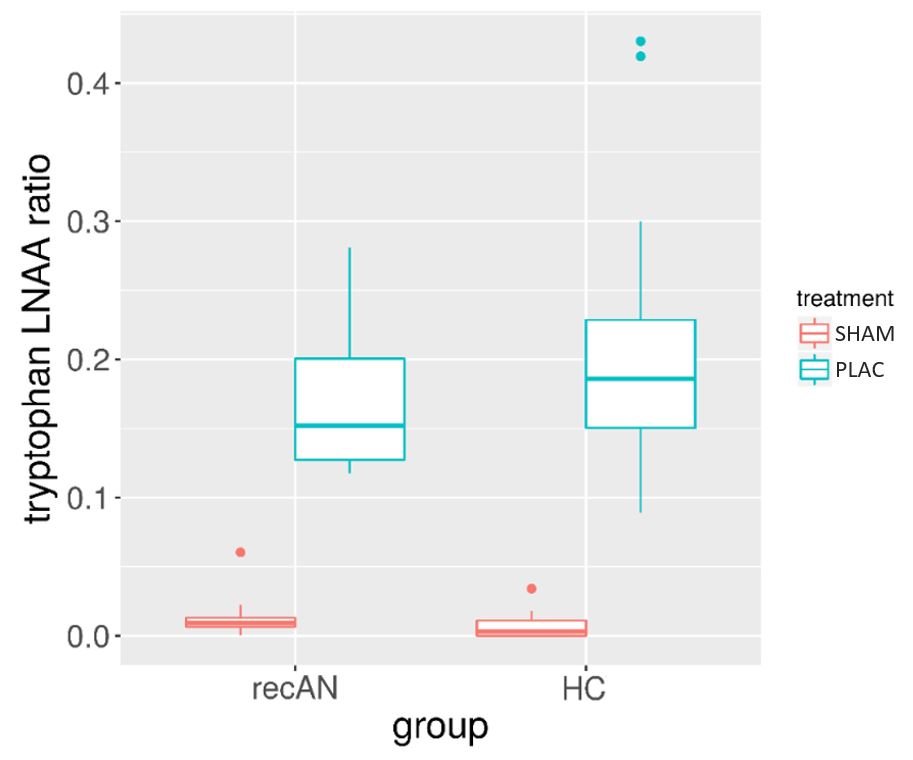


Figure S.1. Boxplots of plasma tryptophan ratios of each group for both conditions showing the successful treatment effect of the acute tryptophan depletion. LNAA=long neutral amino acids, recAN=patient recovered from anorexia nervosa, HC=healthy control participant, DEPL=depletion condition, SHAM=sham depletion.

## 8. Behavioral mixed models

| **Table S.1.** Results of the linear mixed-effects models of the behavioral data. | | | |  |
| --- | --- | --- | --- | --- |
| dependent variable | parameter | F statistics | p value | |
| #bp | reward level | 104.48 | <0.001 | |
|  | group | 0.08 | 0.785 | |
|  | depletion condition | 13.47 | <0.001 | |
|  | group x depletion condition | 0.01 | 0.905 | |
|  | group x reward level | 0.30 | 0.586 | |
|  | depletion condition x reward level | 0.01 | 0.910 | |
|  | group x depletion condition x reward level | 0.03 | 0.865 | |
|  |  |  |  | |
| RT | reward level | 70.02 | <0.001 | |
|  | group | 0.84 | 0.364 | |
|  | depletion condition | 0.72 | 0.395 | |
|  | group x depletion condition | 1.02 | 0.313 | |
|  | group x reward level | 0.14 | 0.707 | |
|  | depletion condition x reward level | 3.00 | 0.084 | |
|  | group x depletion condition x reward level | 0.49 | 0.482 | |
| *Notes.* #bp=number of button presses, RT=reaction times. | | | |  |

## 9. Neuroimaging mixed models

| **Table S.2.** Results of the linear mixed-effects models of the extracted beta values of the brain regions showing three-way interactions using neuropointillist. | | | |
| --- | --- | --- | --- |
| dependent variable | parameter | F statistics | p value |
| left insula | reward level | 27.94 | <0.001 |
| (anticipation) | group | 0.36 | 0.553 |
|  | depletion condition | 0.13 | 0.716 |
|  | group x depletion condition | 0.15 | 0.700 |
|  | group x reward level | 0.24 | 0.626 |
|  | depletion condition x reward level | 1.84 | 0.176 |
|  | **group x depletion condition x reward level** | **22.80** | **<0.001** |
|  |  |  |  |
| right insula | reward level | 10.75 | 0.001 |
| (anticipation) | group | 0.62 | 0.437 |
|  | depletion condition | 0.04 | 0.846 |
|  | group x depletion condition | 0.06 | 0.812 |
|  | group x reward level | 0.19 | 0.665 |
|  | depletion condition x reward level | 1.79 | 0.182 |
|  | **group x depletion condition x reward level** | **15.24** | **<0.001** |
|  |  |  |  |
| mOFC | reward level | 82.23 | <0.001 |
| (feedback) | group | 0.69 | 0.411 |
|  | depletion condition | 0.24 | 0.622 |
|  | group x depletion condition | 0.17 | 0.679 |
|  | group x reward level | 0.98 | 0.324 |
|  | depletion condition x reward level | 7.74 | 0.006 |
|  | **group x depletion condition x reward level** | **16.06** | **<0.001** |
| *Notes.* mOFC=medial orbitofrontal cortex. Please note that the linear mixed-effects models are based on the extracted beta values of the significant three-way interaction clusters obtained with neuropointillist (see Methods section). As a result, only the three-way interactions are of interest (highlighted in bold) while the other main effects and two-way interactions are reported for the sake of completeness. | | | |

**10. Main effect of reward during the anticipation phase**

As a proof of concept, we generated a statistical parameter map of the main effect of reward using neuropointillist and focused on the ventral striatum as one of the main regions of the reward system.


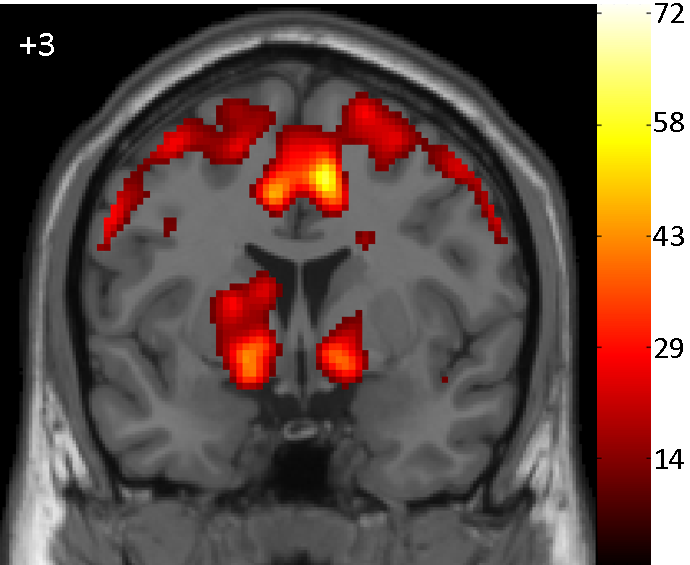


**Figure S.2.** Depiction of the main effect of reward during reward anticipation showing both the left and the right ventral striatum activated as one of the key regions of the reward system. Coronal slice at y=3 (MNI coordinates).

**11. Post-hoc t-tests of the contrasts of the three-way interactions of the linear mixed models**

To investigate whether the recAN group during the sham depletion is mainly contributing to the three-way interactions and whether the ATD condition is causing a normalization of the neural response patterns, we conducted post-hoc Student’s t-tests of the reward level slopes. Namely, as contrasts, we chose to compare (1) the recAN during both conditions, (2) the recAN and the HC during the sham depletion and (3) recAN during ATD with HC during sham.

| **Table S.3.** Results of the post-hoc t-tests. | | | |
| --- | --- | --- | --- |
| ROI, phase | contrast | t statistics | p value |
| left insula, anticipation phase | recAN sham x recAN depletion | 3.17 | 0.005 |
|  | recAN sham x HC sham | 2.40 | 0.021 |
|  | recAN depletion x HC sham | 1.76 | 0.085 |
|  |  |  |  |
| right insula, anticipation phase | recAN sham x recAN depletion | 3.81 | 0.001 |
|  | recAN sham x HC sham | 2.85 | 0.007 |
|  | recAN depletion x HC sham | 1.11 | 0.272 |
|  |  |  |  |
| mOFC, feedback phase | recAN sham x recAN depletion | 3.15 | 0.005 |
|  | recAN sham x HC sham | 2.15 | 0.037 |
|  | recAN depletion x HC sham | 1.51 | 0.139 |
|  |  |  |  |
| *Notes.* For the post-hoc comparisons, we employed Student’s t-tests. ROI=region of interest, HC=healthy control participants, recAN=patients recovered from anorexia nervosa. mOFC=medial orbitofrontal cortex. sham=sham depletion. | | | |

**12. Exploratory correlational analyses**

| **Table S.4.** Exploratory correlational analyses of the BOLD signal slopes during sham depletion in the recAN group. | | | | | | | | |
| --- | --- | --- | --- | --- | --- | --- | --- | --- |
|  | left insula | |  | right insula | |  | mOFC | |
|  | r | p |  | r | p |  | r | p |
| age | .11 | .627 |  | .14 | .53 |  | -.01 | .983 |
| BMI-SDS | -.16 | .465 |  | -.22 | .33 |  | -.15 | .506 |
| BDI-II | -.11 | .613 |  | -.33 | .129 |  | -.01 | .953 |
| EDI total | -.08 | .726 |  | -.19 | .407 |  | -.19 | .405 |
| *Notes.* BOLD=blood-oxygen level dependent. recAN=patients recovered from anorexia nervosa. mOFC=medial orbitofrontal cortex. BMI-SDS=body mass index-standard deviation score. BDI-II=Beck Depression Inventory-II. EDI total=Eating Disorder Inventory - total score. | | | | | | | | |

Following the suggestion of a reviewer, we also ran exploratory correlational analyses between the TRP/LNAA ratio and BMI-SDS (r=-.004, p=.988), the BDI-II (r=-.146, p=.516) and the EDI-2 total score (r=-.312, p=.158). The results were all non-significant.

**References**

Ashburner, J. (2007). A fast diffeomorphic image registration algorithm. *NeuroImage*, *38*(1), 95–113. https://doi.org/10.1016/j.neuroimage.2007.07.007

Bühler, M., Vollstädt-Klein, S., Kobiella, A., Budde, H., Reed, L. J., Braus, D. F., … Smolka, M. N. (2010). Nicotine Dependence Is Characterized by Disordered Reward Processing in a Network Driving Motivation. *Biological Psychiatry*, *67*(8), 745–752. https://doi.org/10.1016/j.biopsych.2009.10.029

Dingerkus, V. L. S., Gaber, T. J., Helmbold, K., Bubenzer, S., Eisert, A., Sánchez, C. L., & Zepf, F. D. (2012). Acute tryptophan depletion in accordance with body weight: Influx of amino acids across the blood–brain barrier. *Journal of Neural Transmission*, *119*(9), 1037–1045. https://doi.org/10.1007/s00702-012-0793-z

Ehrlich, S., Geisler, D., Ritschel, F., King, J., Seidel, M., Boehm, I., … Kroemer, N. (2015). Elevated cognitive control over reward processing in recovered female patients with anorexia nervosa. *Journal of Psychiatry & Neuroscience*, *40*(5), 307–315. https://doi.org/10.1503/jpn.140249

King, J. A., Korb, F. M., Vettermann, R., Ritschel, F., Egner, T., & Ehrlich, S. (2019). Cognitive overcontrol as a trait marker in anorexia nervosa? Aberrant task- and response-set switching in remitted patients. *Journal of Abnormal Psychology*, *128*(8), 806–812. https://doi.org/10.1037/abn0000476

Kroemer, N. B., Guevara, A., Ciocanea Teodorescu, I., Wuttig, F., Kobiella, A., & Smolka, M. N. (2014). Balancing reward and work: Anticipatory brain activation in NAcc and VTA predict effort differentially. *NeuroImage*, *102*, 510–519. https://doi.org/10.1016/j.neuroimage.2014.07.060

Moja, E. A., Stoff, D. M., Gessa, G. L., Castoldi, D., Assereto, R., & Tofanetti, O. (1988). Decrease in plasma tryptophan after tryptophan-free amino acid mixtures in man. *Life Sciences*, *42*(16), 1551–1556. https://doi.org/10.1016/0024-3205(88)90013-6

Richard, D. M., Dawes, M. A., Mathias, C. W., Acheson, A., Hill-Kapturczak, N., & Dougherty, D. M. (2009). L-Tryptophan: Basic Metabolic Functions, Behavioral Research and Therapeutic Indications. *International Journal of Tryptophan Research*, *2*, IJTR.S2129. https://doi.org/10.4137/IJTR.S2129

Zepf, F. D., Sánchez, C. L., Biskup, C. S., Kötting, W. F., Bubenzer, S., Helmbold, K., … Kuhn, C. M. (2014). Acute tryptophan depletion—Converging evidence for decreasing central nervous serotonin synthesis in rodents and humans. *Acta Psychiatrica Scandinavica*, *129*(2), 157–159. https://doi.org/10.1111/acps.12215
